# Register and biobank on influence of assisted reproduction on pregnancy, maternal and neonatal outcome: A prospective multicenter medical data register and biobank

**DOI:** 10.1007/s00508-025-02626-3

**Published:** 2025-10-07

**Authors:** Harald Zeisler, Florian Heinzl

**Affiliations:** Department of Obstetrics and Gynecology, Medical University Vienna, Währinger Gürtel 18–20, 1090 Vienna, Austria

**Keywords:** Assisted reproduction technology, Register, Obstetrical und neonatal adverse outcome, Male factor, Female factor

## Abstract

**Background:**

Infertility affects 10–15% of couples worldwide. In Austria, the In Vitro Fertilization Fund supports assisted reproductive technology under specific conditions. While assisted reproductive technology is generally effective, its impact on pregnancy and neonatal outcomes remains under discussion.

**Methods:**

The Register and Biobank on the Influence of Assisted Reproduction on Pregnancy, Maternal and Neonatal Outcomes is a prospective multicenter register and biobank. It includes five in vitro fertilization centers and two obstetric departments in Vienna and Lower Austria. Standardized clinical and laboratory data are collected via electronic case report forms and pseudonymized using a unique code linking fertility and obstetric data. Recruitment started in 2022, and biobanking (blood, urine, placenta, and umbilical cord samples) was added in 2025.

**Results:**

By early 2025, complete data were available for 366 patients. Male infertility was the most frequent indication (231 cases), followed by tubal factor infertility, polycystic ovary syndrome, and endometriosis. The register enables linking of assisted reproductive technology procedures with detailed pregnancy and neonatal outcomes such as Apgar scores, delivery mode, and Neonatal Intensive Care Unit transfers.

**Conclusion:**

This register offers a unique real-world dataset connecting assisted reproductive technology treatment with maternal and neonatal outcomes. Continued recruitment will support hypothesis-driven research and improve assisted reproductive technology practices. It underlines the importance of high-quality, longitudinal data to understand potential assisted reproductive technology-related risks and guide future care.

## Introduction

Traditionally, infertility has been defined as the failure to achieve a desired pregnancy despite having regular, unprotected sex over a period of at least 1 year [1]; however, the 2023 definition by the American Society for Reproductive Medicine (ASRM) goes far beyond this, attempting to reflect daily reality. For instance, it considers the inability to conceive due to personal circumstances, such as being in a same-sex relationship [2]. Worldwide, around 80 million people, or 10–15% of couples, suffer from infertility; however, the specific prevalence varies depending on the region, and the results of different studies vary [3, 4]. A 2022 survey in Austria revealed prevalence rates of 11% for men and 15% for women, with clear upward shifts according to age [5]. A World Health Organization (WHO) study from 1992 reported a female infertility rate of 37%, a male factor infertility rate of 8%, and a combined rate of 35% [6]; however, a recent publication shows a different distribution pattern: female factor 35–50%, male factor 40–50%. Approximately 85% of infertile couples have an identifiable cause; however, the so-called unexplained infertility rate varies from 15–30% [7, 8]. There are a variety of reasons for female and male infertility. The Register and Biobank of the Influence of Assisted Reproduction on Pregnancy, Maternal and Neonatal Outcomes (AROS) project focuses on four conditions and couples with any of these conditions are financially supported by the In Vitro Fertilization (IVF) Fund of the Austrian Federal Ministry for Labour, Social Affairs, Health and Consumer Protection (BMSGPK). Either the woman must be sterile due to a tubal issue (e.g. blocked fallopian tubes), endometriosis or polycystic ovary syndrome, or the man must have a pathological spermogram. When an IVF fund trial is carried out, the couple must also report the outcome of the trial and any birth to the IVF center that carried out the trial within 3 months [9]. The BMSGPK IVF Fund operates an IVF registry which is maintained as an online application. The IVF centers enter standardized data records for each IVF fund attempt. These data are used to check the eligibility of fertility couples and bill the IVF Fund to IVF centers and to evaluate all IVF Fund attempts as a whole, as well as separately for public and private IVF centers. Additionally, the pregnancy and baby take-home rates, as well as the mode and weeks of gestation at delivery, are recorded; however, these data are generally not collected for research purposes [10].

Medically assisted reproduction (MAR) involves the use of medical methods to achieve pregnancy that do not involve sexual intercourse. As no patients undergoing artificial insemination are currently included, the term assisted reproductive technology (ART) is used throughout, which encompasses all treatments or procedures involving the in vitro handling of human oocytes, sperm, or embryos for the purpose of establishing a pregnancy [11]. The use of ART has increased steadily in recent years for various reasons. The indications for the use of ART have expanded, and most individuals undergoing ART and their resulting offspring are healthy; however, ART has been associated with an increased risk of adverse pregnancy and maternal outcomes [12]. It is a complex process characterized by rapid technological advances in ART performance. In AROS, the course of pregnancy, delivery and maternal and offspring outcomes for couples who have undergone fertility treatment will be recorded prospectively and made available for descriptive evaluation. There is an increasing need to distinguish between the effects of ART on outcomes and those of other confounding or mediating factors. The aim of AROS is to provide clinicians and researchers with data on current fertility practices and the effectiveness and safety of ART treatment as well as forming the basis for future studies and optimized management.

## Patients, material and methods

In 2018, the Ethics Committee of the Medical University of Vienna gave AROS the green light; however, due to the coronavirus 2019 pandemic, recruitment could not begin until 2022. Starting as a register, AROS expanded into a biobank in 2025. Representing a prospective multicenter medical data register and biobank, AROS is based in Vienna, the capital of Austria and five IVF centers in Vienna and two in Lower Austria participate in AROS, as do the Department of Obstetrics and Gynecology at the Medical University of Vienna, General Hospital Vienna and the University Hospital Tulln and the Department of Obstetrics and Gynecology at the Karl Landsteiner Private University of Health Sciences. Recruitment is still ongoing. Each study project using data from the register/biobank must be submitted separately to the Ethics Committee of the Medical University of Vienna. At the beginning of 2025, around 3.7 million people were living in Vienna and Lower Austria, accounting for 40.9% of the total Austrian population [13].

In a real-world cohort, patients are treated at participating centers according to local standards and guidelines. Admission to AROS does not influence the treatment of referred patients. Participants are women who registered for childbirth at the Department of Obstetrics and Gynecology at the Medical University of Vienna, General Hospital of Vienna, and the University Hospital of Tulln and the Department of Obstetrics and Gynecology at the Karl Landsteiner Private University of Health Sciences. The women are divided into two groups: those who have undergone fertility treatment and those who have conceived spontaneously, the latter group being included for comparison purposes with respect to the biobank.

The inclusion and exclusion criteria are shown in Table 1.

**Table 1:** Inclusion and exclusion criteria

Inclusion criteria	Written informed consent
	Age ≤ 40 years for women and ≤ 50 years for men in accordance with the legal requirements of the IVF Fund
	Fertility treatment in one of the participating fertility centers
	Couples with spontaneous conception
	Baby take home
Exclusion criteria	None

For the AROS study, routine clinical information is collected from the medical records of participating patients and transferred directly into internet-based electronic case report forms (eCRFs). Data entry sheets for “Obstetrics” and “Assisted Reproduction” from SCICOMED e. U. (www.scicomed.net) are used for this purpose (governed by a framework and data protection agreement with the Medical University of Vienna). The company aims to provide the highest quality database solutions. The data documentation sheets were developed in collaboration with experts and are continuously adapted in line with the latest findings. A special feature is the high level of user-friendliness. The database solutions have the following special features: English language for international use:

- Quick information bar for a quick overview, drop-down lists to avoid typing errors; however, the major innovation is merging the two databases using an individualized code called “Share Code”. This code is generated in the Assisted Reproduction database. It is entered into a special input field in the obstetrics database to create the link. When a patient registers for childbirth or gives birth at the Department of Obstetrics and Gynecology, they must sign the patient information and consent form, which allows data from both obstetrics and fertility treatment to be used. Each participating center receives a user name and password, giving them sovereignty over the entered data set. The AROS management committee consists of the study investigators from all participating centers and is chaired by the AROS team from the Department of Obstetrics and Gynecology at the Medical University of Vienna. The AROS team consists of a principal investigator, a scientific co-worker and a statistician. The principal investigator at the Department of Obstetrics and Gynecology in Vienna has the right to veto the use of the consolidated dataset. This procedure is regulated by a detailed contract between the Medical University Vienna and participating centers.

Once participants have signed the informed consent form, the AROS team accesses their medical health records from the IVF centers and hospitals. Patient information is collected from the first consultation on the postpartum ward, using a physical documentation sheet identical to the eCRF in the Obstetrics database solution from SCICOMED e. U. This sheet contains a detailed anamnesis. The pseudonymization process begins by assigning a separate code to each participant. This code is generated automatically as soon as the first data are entered into the SCICOMED database. The code, the number of participants recruited and their personal data, such as family name, first name, date of birth, date of inclusion, estimated date of delivery and name of the IVF center, are recorded in a special file. Only the AROS team has access to this file. The storage medium is kept in a safe. A file is also created for each IVF center, which is managed jointly by the AROS team and the IVF center. For quality assurance purposes, data from the IVF centers are entered into SCICOMED e. U. together with the AROS team. Another quality assurance measure is that the AROS data are validated annually for accuracy, veracity and integrity by the AROS team.

**Fig. 1:**
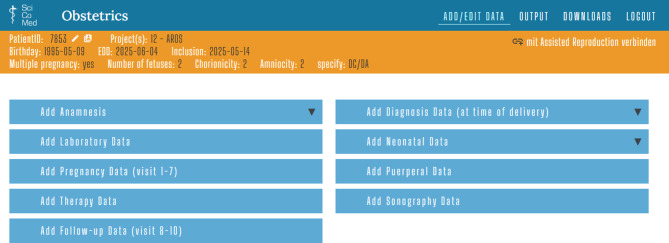
Input screen for the Obstetrics database solution with the corresponding input sheets

The Obstetrics database solution comprises comprehensive obstetric documentation sheets. These include anamnesis data, diagnosis data (at the time of delivery), laboratory data, neonatal data, pregnancy data (visits 1–7), puerperal data, therapy data, sonography data and follow-up data (visits 8–10). In the context of biobanking, i.e. the simultaneous collection of samples and data, up to 10 measuring points can be created; however, the data documentation sheets can also be used for any obstetric subproject. For AROS, only a small selection is used, namely anamnesis data, diagnosis data, neonatal data, and puerperal data (Fig. 1 and Tab. 3).

Table 2 shows all the data variables obtained from the anamnesis, organized by category.

**Table 2:** Variables collected on demographics and medical and obstetric history

Variable	Value
Age	Years
Height	cm
Weight	kg
Ethnicity	Europeans of different ethnicities, African, Hispanic, Asian, Other
Religious confession	Roman Catholic, Protestant, Orthodox, Islamic, Jewish, Buddhist, Hindu, other, without religion belief
Highest level of education	Primary school, lower secondary school, special school, polytechnic course, vocational school and apprenticeship, vocational secondary school, general school (upper secondary level), vocational secondary school, academies and universities of applied sciences, university
Relationship status	Single, in partnership, married
Smoker	Non-smoker, ex-smoker, smoker
Alcohol	Yes, no
Drugs	Yes, no
Pregnancy	Number
Birth	Number
Conception	Spontaneous, assisted reproduction technology (ART)
Estimated day of delivery (EDD)	Date
Abortion	Yes, no
Ectopic pregnancy	Yes, no
Stillbirth	Yes, no
Birth	Number
Premature birth	Number
Cesarean section	Number
Previous pregnancy diseases	Diabetes, hypertensive disorder
Previous illnesses	Type
Previous surgeries	Type

**Fig. 2:**
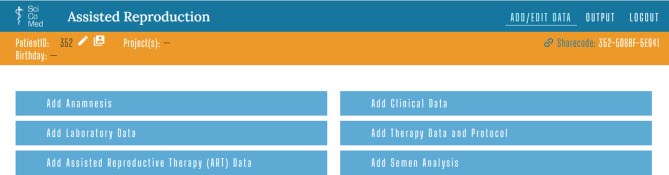
Shows the input screen for the Assisted Reproduction database solution, along with the corresponding input sheets

The ART encompasses all procedures involving the in vitro manipulation of human oocytes, sperm, or embryos for reproductive purposes. This encompasses far more than just IVF and embryo transfer (ET), or intracytoplasmic sperm injection (ICSI). The birth of Louise Brown in 1978, the first baby conceived through ART, marked a milestone in reproductive medicine and fulfilled countless wishes for children [14]. Since then at least 12 million children have been born worldwide through artificial insemination [15]. In Austria alone, this resulted in 3535 pregnancies in 2022 [16]. The development of ART has been driven by numerous innovations. These include improvements in stimulation protocols as well as the optimization of sperm preparation and embryo culture methods. Cryopreservation, which enables embryos to be stored safely and kept ready for future use if required, has also been a significant development [17]. The Assisted Reproduction database solution enables all steps of fertility treatment to be recorded, including ART cycles, anamneses, clinical data, laboratory data, therapy data, protocols and ART and semen analyses. The last four are used for AROS (see Fig. 2 and Table 4).

**Table 3:** All obtained data variables of the diagnosis, neonatal outcomes and puerperal outcomes organized by category

Variable	Value
Diagnosis data	
Loss of follow-up data	Yes, no
Child number	Number
Day of delivery	Number
Pregnancy summarization	Uneventful pregnancy, complications in pregnancy
Mode of delivery	Vaginal delivery vacuum/forceps, cesarean section elective, primary, secondary, emergency, induced/therapeutic abortion
Induction of labor	Yes, no
Anesthesia	No, epidural, spinal, general
Diagnosis/indication	Pregnancy-related maternal diseases: hypertensive disorder, gestational diabetes mellitus
	Fetal and pregnancy-related diseases and complications: breech presentation, fetal distress, abnormal Doppler ultrasound, intrauterine growth retardation (IUGR), small for gestational-age (SGA), large for gestational-age (LGA), stillbirth, fetal malformation, placenta previa, prolonged pregnancy, preterm rupture of membranes (PROM), preterm labor (PTL), previous cesarean section, others
Neonatal data	
Birthweight/percentile	Number
Major fetal malformation	Yes, no
Apgar score 1, 5, 10	Number
Arterial pH value (ArtpH), Base excess (BE)	Number
Transfer to NICU	Yes, no
Perinatal mortality	Yes, no
Neonatal morbidity	Yes, no
	Respiratory distress syndrome (RDS), bronchopulmonary dysplasia (BPD), surfactant, ventilation, continuous positive airway pressure (CPAP), intubation, oxygen supply, necrotic enterocolitis, intraventricular hemorrhage (IVH), periventricular leukomalacia (PVL), catecholamines, sepsis, others
Puerperal data	
Hospitalization days	Number
Revision, fever, mastitis, endometritis, thrombosis/embolism, transfusion, intensive care unit	Yes, no

Table 4 shows the input sheets currently used in AROS. Medical history is taken from the Obstetrics database. While clinical data would be of interest in connection with endometriosis, for example, it is not currently recorded.

**Table 4:** List of possible parameters in the data sheets laboratory data, ART data, therapy data/protocol and semen analysis in Assisted reproduction database

Laboratory data	
Variable	Value
Date of sampling	Number
Day of menstrual cycle	Number
Beta human chorionic gonadotropin (HCG) (U/l)	Number
Thyroid-stimulating hormone (TSH) (mU/l)	
free T3 (triiodothyronine) (pg/ml)	
free T4 (thyroxine) (ng/ml)	
Estradiol (pg/ml)	
Estriol (µg/l)	
Testosterone (ng/ml)	
Dehydroepiandrosterone (DHEA) (ng/dl), Dehydroepiandrosterone sulfate (DHEA-S) (µg/dl), androstenedione (ng/ml)	
Dihydrotestosterone (DHT) (ng/dl)	
Aldosterone (ng/l)	
11-Deoxycortisol (nmol/l)	
Cortisol serum (µg/dl)	
17-Hydroxyprogesterone (ng/ml)	
Sex hormone-binding globulin (SHBG) (nmol/l)	
Progesterone (ng/ml)	
Luteinizing hormone (LH) (mU/ml)	
Follicle stimulating hormone (FSH) (mU/ml)	
Anti-Müllerian hormone (AMH) (ng/ml)	
Vitamin D (IU)	
oral glucose tolerance test (oGTT)	
Fasting insulin (mol/l)	
Homeostasis Model Assessment (HOMA) index	
Prolactin (mU/l)	
Others	
ART data	
Variable	Value
ART	IVF, ICSI, cryotransfer natural cycle, cryotransfer drug supported
Transfer	Date
	Egg and/or sperm donation
	Single, double or other embryo transfer
	Single, double or other blastocyte transfer
Outcome	Pregnancy test (urine)-positive/negative, human chorionic gonadotropin (HCG), progesterone
	Date of ultrasound, intrauterine, extrauterine, gestational sack, yolk sack, fetal pole, heartbeat positive, crown-rump length, baby take home
Therapy data and protocol	
Variable	Value
Protocol	No, timed intercourse natural cycle, timed intercourse stimulated cycle, intrauterine insemination (IUI), natural cycle intrauterine insemination (IUI), stimulated cycle in vitro fertilization (IVF), intracytoplasmic sperm injection (ICSI), cryotransfer natural cycle, cryotransfer drug supported cycle
ART-related medication	
Duration of stimulation	Number
Day of egg collection	Number
Egg retrieval procedure	Vaginal sonography, other technical procedure
Number of oocytes, number of maturated oocytes, number of fertilizations, cryopreservation	Number
Semen analysis	
Variable	Value
Semen volume (ml), total sperm number (10^6^ per ejaculation), sperm concentration (10^6^ per ml), total motility (PR + NR, %), progressive motility (PR, %), vitality (live spermatozoa, %), sperm morphology (normal forms, %), pH, peroxidase positive leucocytes (10^6^ per ml), MAR test (motile spermatozoa with bound particles, %), immunobead test (motile spermatozoa with bound beads, %), seminal zinc (µmol/ejaculate), seminal fructose, seminal neutral glucosidase (mU/ejaculate)	Number
Result	

As part of fertility treatment, IVF centers follow either the current WHO criteria for the spermogram or a special agreement with the IVF Fund. The threshold values are shown in Table 5. A private contract between the IVF centers and the IVF Fund regulates these special criteria. This information is not publicly available; it is derived from data provided by the IVF centers [18, 19].

**Table 5:** Threshold values according to WHO Manual, 6th edition [18] and IVF Fund criteria [19]

	WHO	IVF Fund
Sperm concentration (million/ml) (previously 15 ml)	≥ 16	≥ 10
Total sperm count per ejaculate (million)	≥ 39	–
*Motility (%)*		
Forward-moving sperm	≥ 30	≥ 20
Total motility: ≥ 42% motile	≥ 42	–
Morphology (%) normally shaped sperm	≥ 4	≥ 30

If the relevant requirements are met, the IVF Fund will cover 70% of the ART costs for up to 4 attempts. This means that the costs of IVF, ICSI, cryotransfer, and the collection of sperm from the testicles (TESE) or the epididymis (MESA) will be largely covered. At the start of the first attempt, the woman intending to carry the child to term must not have reached the age of 40 years (40th birthday) and her partner must not have reached the age of 50 years (50th birthday). A specialist in gynecology and obstetrics must make the following diagnoses:

- Tubal factor: presence of bilaterally blocked or otherwise permanently nonfunctional fallopian tubes, as detected by an imaging or surgical procedure.
- Endometriosis: presence of endometriosis as detected by an imaging (ultrasound or MRI) or surgical (including histology) procedure, resulting in functional sterility.
- PCOS: presence of polycystic ovaries, as detected by an imaging procedure; chronic anovulation; and functional sterility, after excluding other endocrine disorders. A specialist (e.g. a urologist) must diagnose male infertility: sterility or severe male infertility with an underlying medical diagnosis, or idiopathic pathospermia (without a causal medical diagnosis) with the presence of two pathological spermograms performed at least 4 weeks apart [19].

The register has been expanded to include biobanking, meaning that blood and tissue samples can now be collected. Participants are informed that samples are either taken as part of a diagnostic and/or therapeutic procedure or used exclusively for AROS purposes. Consent applies to both retrospective and prospective projects. The principle of proportionality is applied with respect to sampling and the amount of material collected at each stage.

Material collection:

Blood samples (3 × 9 ml) and urine samples (4 × 1.5 ml) are taken from both female and male participants undergoing fertility treatment, or only from women during pregnancy at each appointment. Umbilical cord blood (arterial and venous) and placenta (1/4 at the base of the umbilical cord, including part of the umbilical cord) are collected after birth. No blood is taken from healthy newborns as umbilical cord blood is available for this purpose. In the case of sick newborns, blood samples are only taken as part of a planned sample for diagnostic purposes or as part of treatment. An additional quantity of up to 1 ml is taken for this purpose. For premature newborns, the maximum quantity is 150µl.

## Results

**Fig. 3:**
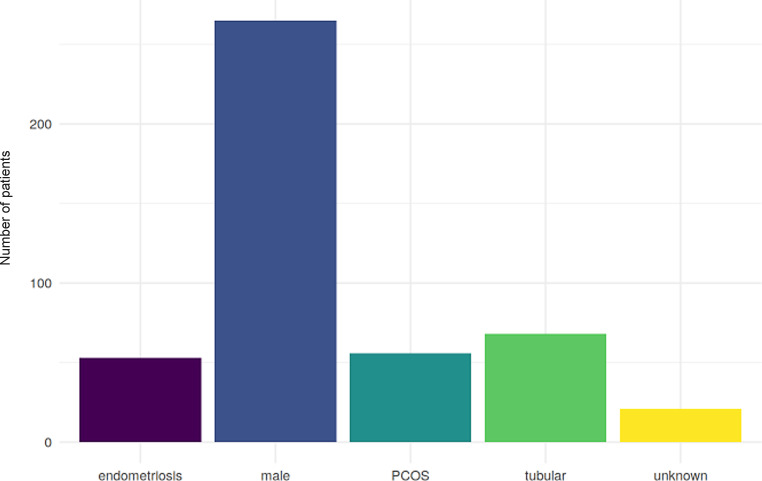
Graphical representation of the frequency of the indications according to FONDS criteria

**Fig. 4:**
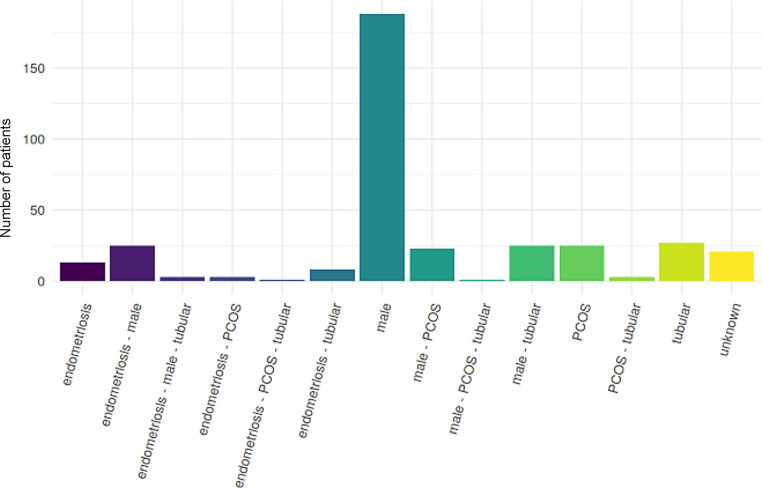
Graphical representation of the frequency of combinations of the indications according to FONDS criteria

Currently, the database contains complete datasets for a total of 366 patients. Of the four most prominent factors (endometriosis, tubal factor, PCOS and male factor), male factor was the single most prevalent (265 out of 366 patients according to IVF Fund criteria and 231 according to WHO), with tubal factor the second most prevalent factor (68 cases). A PCOS was observed in 56 patients and endometriosis in 53. In 21 cases, no factors could be identified. When these factors were combined into a single variable, the most common factor was still male (188), with tubal factor in second place (see Table 5, 6 and 7 and Figs. 3 and 4).

**Table 6:** List of the frequency of the indications according to IVF Fund criteria

Type	Count
Male	265
Tubal factor	68
PCOS	56
Endometriosis	53
Unknown	21

**Table 7:** List of the frequency of combinations of the indication according to IVF Fund criteria

Combined	Count
Male	ISS
Tubal factor	27
Endometriosis—male	25
Male—tubal factor	25
PCOS	25
Male—PCOS	23
Unknown	21
Endometriosis	13
Endometriosis—tubal factor	S
Endometriosis—male—tubal factor	3
Endometriosis—PCOS	3
PCOS—tubal factor	3
Endometriosis—PCOS—tubal factor	1
Male—PCOS—tubal factor	1

The software used for the statistical analysis was R version 4.5.0 [20–22].

## Discussion

The Register and Biobank of Influence of Assisted Reproduction on Pregnancy, Maternal and Neonatal Outcome (AROS) is a prospective, multicenter data register and biobank designed to investigate the implications of assisted reproductive technology (ART) on maternal and neonatal health outcomes. The AROS currently incorporates comprehensive data from five IVF centers and two hospitals in Austria. Methodologically, AROS integrates standardized clinical and laboratory data from fertility treatment and obstetric care using a pseudonymized internet-based electronic data capture system. This dual-database approach enables linkage between reproductive history and pregnancy outcomes. To date, a total of 366 patients with complete datasets have been analyzed, revealing that male infertility is the most frequent indication for ART, followed by tubal factor, PCOS and endometriosis. Developing a structured, high-quality scientific database such as AROS is essential in a field characterized by rapidly evolving technologies and practices. It enables long-term tracking, quality assurance and generation of evidence regarding ART outcomes. The biobank component further enhances the potential for future research by enabling biomolecular studies to be conducted alongside clinical evaluations. Looking ahead, AROS is set to become an invaluable resource for clinicians and researchers alike, providing a robust foundation for hypothesis-driven studies and the optimization of ART protocols. As the dataset grows, it will enable more detailed analyses of maternal and neonatal risks, inform policy development and guide patient counselling, ultimately contributing to improved reproductive care and health outcomes.

## Abbreviations

AMH: Anti-Müllerian hormone
ART: Assisted reproductive technology
ASRM: American Society for Reproductive Medicine
BMSGPK: Federal Ministry of Labour, Social Affairs, Health Care and Consumer Protection
BPD: Bronchopulmonary dysplasia
CPAP: Continuous positive airway pressure
DHEA: Dehydroepiandrosterone
DHEA‑S: Dehydroepiandrosterone sulphate
DHT: Dihydrotestosterone
EDD: Estimated day of delivery
ET: Embryo transfer
FSH: Follicle-stimulating hormone
HCG: Human chorionic gonadotropin
HOMA: Homeostasis model assessment
ICSI: Intracytoplasmic sperm injection
IUGR: Intrauterine growth retardation
IUI: Intrauterine insemination
IVF: In vitro fertilization
IVF fund: Fund for the financing of in vitro fertilization
IVH: Intraventricular hemorrhage
LGA: Large for gestational age
LH: Luteinizing hormone
MAR: Medically assisted reproduction
MART: Mixed antiglobulin reaction test
MESA: Microsurgical epididymal sperm aspiration
NICU: Neonatal intensive care unit
PCOS: Polycystic ovary syndrome
PROM: Premature rupture of membranes
PTL: Preterm labor
PVL: Periventricular leukomalacia
RDS: Respiratory distress syndrome
SHBG: Sex hormone binding globulin
TSH: Thyroid-stimulating hormone
WHO: World Health Organization
